# Conducting Eating Disorder Research in the Era of Generative AI: Researcher Perspectives and Guidelines From the *International Journal of Eating Disorders*

**DOI:** 10.1002/eat.24543

**Published:** 2025-09-07

**Authors:** Jake Linardon, Jennifer J. Thomas, Scott J. Crow, Ata Ghaderi, Anja Hilbert, Kelly L. Klump, Tracey D. Wade, B. Timothy Walsh, Ruth Weissman

**Affiliations:** ^1^ SEED Lifespan Strategic Research Centre, Faculty of Health, School of Psychology Deakin University Geelong Victoria Australia; ^2^ Eating Disorders Clinical and Research Program Massachusetts General Hospital Boston Massachusetts USA; ^3^ Department of Psychiatry Harvard Medical School Boston Massachusetts USA; ^4^ Department of Psychiatry and Behavioral Sciences University of Minnesota Minneapolis Minnesota USA; ^5^ Division of Psychology, Department of Clinical Neuroscience Karolinska Institutet Stockholm Sweden; ^6^ Integrated Research and Treatment Center Adiposity Diseases, Behavioral Medicine Research Unit, Department of Psychosomatic Medicine and Psychotherapy Leipzig University Medical Centre Leipzig Germany; ^7^ Department of Psychology Michigan State University East Lansing Michigan USA; ^8^ Flinders University Institute for Mental Health and Wellbeing Flinders University Adelaide South Australia Australia; ^9^ Department of Psychiatry Columbia University Irving Medical Center New York New York USA; ^10^ New York State Psychiatric Institute New York USA; ^11^ Department of Psychology Wesleyan University Middletown Connecticut USA

**Keywords:** artificial intelligence, editorial board, feeding and eating disorder, guidelines, large language models, science, survey, technology

## Abstract

**Objectives:**

Generative Artificial Intelligence (AI) could transform how science is conducted, supporting researchers with writing, coding, peer review, and evidence synthesis. However, it is not yet known how eating disorder researchers utilize generative AI, and uncertainty remains regarding its safe, ethical, and transparent use. The Executive Committee of the *International Journal of Eating Disorders* disseminated a survey for eating disorder researchers investigating their practices and perspectives on generative AI, with the goal of informing guidelines on appropriate AI use for authors, reviewers, and editors.

**Method:**

A survey was distributed globally via eating disorder organizations, professional networks, and individual researchers. Researchers (*N* = 158) of various career stages completed the survey.

**Results:**

Nearly three‐quarters (70%) reported using generative AI for research, most commonly for proofreading written work or coding support. Nine in 10 took steps to verify AI‐generated output, and 1 in 3 disclosed their use of AI. Only 21% reported using AI for peer review, typically in a limited capacity (e.g., proofreading), and always with full human oversight. Authors were comfortable for editors to use AI to support administrative tasks (i.e., selecting reviewers, detecting plagiarism). However, many participants acknowledged key drawbacks of generative AI, including concerns about inaccurate outputs, ethical issues such as plagiarism, the potential for reduced critical thinking, and anticipated negative impacts on the future of eating disorder research.

**Conclusion:**

These insights informed the development of field‐specific guidelines to support authors, reviewers, and editors in the appropriate use of generative AI in eating disorder research and publishing.

**Summary**

- 70% of eating disorder researchers use generative AI, primarily for proofreading and coding, with 90% verifying outputs and 33% disclosing AI use.
- Only 21% use AI for peer review, typically for minor tasks like proofreading, with full human oversight.
- Researchers noted risks like inaccuracy, plagiarism, and reduced critical thinking, leading to field‐specific AI use guidelines for authors, reviewers, and editors.

## 1 Introduction

Generative artificial intelligence (AI) is more relevant than ever before, with sectors such as healthcare, education, and finance increasingly integrating these systems into their workflows and decision‐making processes (Ertel 2024; Torous and Blease 2024). Generative AI refers to systems capable of generating new content—including text, images, audio, or code—by learning patterns from large datasets and responding to user prompts (Lv 2023). These tools are capable of drafting text, summarizing documents, generating code, creating visual designs, and supporting idea generation across a range of applications.

Given these capabilities, generative AI tools may be well‐suited to assist with various research‐related tasks that can support authors (e.g., debugging code, structuring manuscripts), reviewers (e.g., proofreading review reports, summarizing submissions), and journal editors (e.g., locating suitable reviewers, detecting plagiarism). This is especially appealing to academics managing high workloads across research, teaching, and service, who are nonetheless expected to produce high volumes of output within short timeframes to remain competitive for academic promotions, funding opportunities, and research recognition (Van Dalen 2021). Emerging evidence suggests that many academics across the physical, social, and life sciences are using generative AI tools to support various aspects of scientific research, but they also express concerns about their limitations and uncertainty around appropriate use, disclosure practices, and ethical implications (Andersen et al. 2025; Linardon et al. 2025; Nicholas et al. 2024; Van Noorden and Perkel 2023).

Given the increasing uptake of generative AI among researchers, alongside ongoing uncertainty regarding its appropriate, ethical, and transparent use (Van Noorden and Perkel 2023), the Executive Committee of the *International Journal of Eating Disorders* (IJED) sought to develop recommendations for authors, reviewers, and journal editors in our field. Although recommendations for generative AI use have been formed for other disciplines (e.g., radiology, medicine; Luo et al. 2025; Moy 2023) or journals (e.g., JMIR; Leung et al. 2023), to our knowledge there is a dearth of such guidelines for mental health research, where sensitive data, vulnerable populations, and nuanced clinical or sociocultural interpretations raise unique challenges. These challenges are not exclusive to eating disorders but are shared across mental health more broadly; by developing field‐specific guidance in eating disorders, we hope this work can serve as a model for other areas should there be a need for disorder‐specific guidelines.

As a foundational step, we conducted a survey exploring how eating disorder researchers are currently using generative AI, the extent and purpose of this use, reasons for non‐use, and their perceptions of its potential benefits, limitations, and future influence on the field. Such data are critical for informing the development of field‐specific guidelines aimed at supporting safe, transparent, and contextually appropriate use of generative AI across the research and publication lifecycle of the eating disorders field.

## 2 Method

### 2.1 Design

A cross‐sectional web‐based anonymous survey was delivered via Qualtrics to eating disorder researchers in June 2025.

### 2.2 Participants and Procedure

Participants were eligible if they were active researchers whose primary focus was eating disorders, defined as having over 50% of their research outputs in this field. This also included research students (PhD, MD, or Doctoral students). While we targeted groups likely to meet these criteria, participants selected themselves into the study based on the eligibility information provided in the survey invitation. Invitations to complete this survey were distributed via email to editorial board members of the: IJED (*N* = 105), European Eating Disorders Review; EEDR (*N* = 28, after removing overlap with the IJED board); Journal of Eating Disorders (*N* = 26, non‐overlapping with the IJED or EEDR); the Eating Disorder Research Society (*N* = 281), and a selected number of corresponding authors of recent publications in the IJED, EEDR, and Eating Disorders: A Journal of Treatment and Prevention (*N* = 150). We also encouraged survey invitation recipients to forward an invitation to other membership groups and active researchers in the field. Study advertisements indicated that the Executive Committee of the IJED was aiming to understand author and reviewer practices and perspectives of generative AI tools in order to inform future editorial policies and guidelines. The survey took about 15 min to complete. No compensation was offered. Given the diverse recruitment methods and follow‐ups, and the unknown number of individuals who received the survey link from colleagues, we could not calculate a response rate.

### 2.3 Measures

The survey was self‐developed for the purposes of this study, informed by prior survey research on generative AI use among researchers in other scientific disciplines (Van Noorden and Perkel 2023), and refined through an iterative process involving review, discussion, and consensus among all members of the IJED Executive Committee to ensure content suitability for the target audience. The full survey is presented in the Supporting Information.

Participants first provided basic demographic (e.g., age, gender, etc.) and academic details (e.g., job title, number of publications, etc.). Before commencing the next phase of the survey, participants were presented with the following lay definition of generative AI: “The following questions ask about your experience with and perspectives towards generative artificial intelligence. Generative AI refers to AI systems that can create new content such as text, images, audio, or code. These tools are trained on large datasets and can generate human‐like responses or original outputs based on a prompt. Common examples include ChatGPT, DALL·E, Midjourney, and GitHub Copilot. Generative AI can assist with tasks like summarizing articles, drafting emails, creating figures, writing code, and generating research ideas.”

Participants indicated whether they had used generative AI tools for research purposes. Those who responded “no” specified their reasons for non‐use among a pre‐selected set of options (e.g., concerns with accuracy or output, data privacy concerns, preference for human‐led thinking, etc.), while those who responded “yes” were then asked to indicate the type of tools used, frequency of use, purpose for use (e.g., drafting research papers, proof‐reading, coding support, etc.), steps taken to verify outputs, disclosure practices of generative AI in publications, and impact of use on their research. Users were also asked whether they had used generative AI tools to assist with peer review tasks. Those who reported “yes” were also asked to indicate how frequently they used these tools and the extent to which they used them for various peer review tasks, ranging from proofreading their review to generating a review independently with minimal or no human oversight.

The entire sample was then presented with a list of potential uses of generative AI by journal editors and peer reviewers (e.g., detecting plagiarism and duplicated content, assisting editors in identifying reviewers, translating reviewer comments into clearer language, etc.) and asked to indicate which uses they would feel comfortable with during the manuscript submission and review process. Then, among a pre‐selected set of options, all participants were asked to select what they thought were the current benefits (e.g., enhanced creativity and hypothesis generation, improved efficiency, supports code generation, etc.) and limitations (e.g., potential for inaccurate information, reduced critical thinking, lack of transparency in outputs, etc.) of generative AI tools for eating disorder research. Finally, all participants were presented with a series of statements about how generative AI may impact the eating disorder field over the next 5 years (e.g., will accelerate the pace of research, will enhance innovation in digital interventions, will complicate the ethical landscape for testing new treatments, etc.), and were asked to indicate their level of agreement on a five‐point scale, ranging from strongly disagree to strongly agree.

### 2.4 Statistical Analysis

Given the descriptive nature of the study, analyses focused on reporting percentages for each survey item to summarize responses on generative AI use, perceptions, and comfort levels.

## 3 Results

### 3.1 Sample Characteristics

One hundred fifty‐eight participants completed the survey (M_age_ = 42.14, SD = 12.92). The majority of participants identified as women, resided in either North America, Oceania, or Europe, and had a PhD/Doctorate degree. Job titles varied, with most reporting to be either a Research Student, Professor, Assistant Professor, Associate Professor, or Clinician–Scientist. The number of publications also varied, with half of the sample reporting less than 60 publications. The most common areas of eating disorder research were treatment/early intervention, etiology, risk factors, and assessment/diagnosis (Table 1).

**TABLE 1:** Sample characteristics (*N* = 158).

Characteristic	Data
Age	42.14 (SD = 12.92)
Gender	
Women	114 (72.2%)
Man	36 (22.8%)
Gender diverse	4 (2.5%)
Did not say	4 (2.5%)
Continent	
North America	80 (50.6%)
Europe	29 (18.4%)
Oceania	35 (22.1%)
Asia	6 (3.8%)
South America	3 (1.9%)
Did not report	5 (3.2%)
Qualification	
Bachelor's degree or equivalent	8 (5.1%)
Master's degree or equivalent	25 (15.8%)
PhD/Doctorate degree	109 (69.0%)
Medical degree	12 (7.6%)
Other	4 (2.5%)
Job title	
Research Student	35 (22.2%)
Post‐Doctoral Researcher	15 (9.5%)
[Senior] Research Fellow	5 (3.2%)
Lecturer/Assistant Professor	15 (9.5%)
Associate Professor	21 (13.3%)
Professor	32 (20.3%)
Research‐Scientist	7 (4.4%)
Clinician–Scientist	21 (13.3%)
Other	7 (4.4%)
Peer‐reviewed publications	
1–20	50 (31.6%)
21–40	23 (14.6%)
41–60	18 (11.4%)
61–80	17 (10.8%)
81–100	14 (8.9%)
101–150	13 (8.2%)
> 150	22 (13.9%)
Not sure	1 (0.6%)
Primary Research Focus	
Treatment/early intervention	103 (65.2%)
Prevention	29 (18.4%)
Neurobiology/neuroimaging	23 (14.6%)
Genetics	12 (7.6%)
Assessment/diagnosis	52 (32.9%)
Epidemiology	24 (15.2%)
Etiology, risk factors, and correlates	86 (54.4%)
Health services and implementation science	34 (21.5%)
Policy and advocacy	10 (6.3%)
Other	14 (8.9%)

*Note*: Participants could select more than one option for the research focus question.

### 3.2 Generative AI Use

When asked whether they had used generative AI to assist with research, 110 (69.6%) responded “yes” and 48 (30.4%) responded “no.”

#### 3.2.1 Reasons for Non‐Use

Among non‐users (*n* = 48), the most common reasons for not using generative AI, in order of endorsement, were “concerns about the accuracy or reliability of output” (*N* = 34; 70.8%), “concerns about plagiarism or academic misconduct” (*N* = 34; 70.8%), “preference for human‐led thinking and writing” (*n* = 32; 66.7%), “concerns about undermining originality and creativity” (*n* = 31; 64.6%), “lack of policies or unclear guidelines around its use” (*n* = 20; 41.7%), “unfamiliarity or lack of consideration” (*n* = 19; 39.6%), “limited time or interest in learning these tools” (*n* = 17; 35.4%), “privacy or confidentiality concerns” (*n* = 17; 35.4%), “accessibility issues” (*n* = 3; 6.3%), and “concerns about reputational risk” (*n* = 2; 4.2%).

### 3.3 Generative AI Users

#### 3.3.1 Tools Used

Among generative AI users (*n* = 110), the most common tool used for research assistance was ChatGPT (*n* = 99; 90%). Other tools used were Microsoft Copilot (*n* = 18; 16.4%), Google's Gemini (*n* = 9; 8.2%), Anthropic's Claude (*n* = 9; 8.2%), Dall‐E (*n* = 8; 7.3%), Perplexity (*n* = 9; 5.7%), and GitHub Copilot (*n* = 4; 3.6%). Sixteen participants (14.5%) selected “other.”

#### 3.3.2 Frequency of Use

When asked to indicate the frequency of generative AI use, 19 participants (17.3%) indicated daily, 42 (38.2%) weekly, 16 (14.5%) monthly, 22 (20.0%) occasionally, and 11 (10.0%) indicated only once or twice.

#### 3.3.3 Purpose of Generative AI Use

Table 2 displays participant endorsements of research tasks for which they used generative AI assistance. The tasks that received the highest endorsement were “proofreading written work” (*n* = 80; 72.7%), “support with coding” (*n* = 46; 41.8%), “writing professional emails or research correspondence” (*n* = 45; 40.9%), “drafting or structuring written work” (*n* = 41; 37.3%), and “preparing lay summaries or audience‐specific materials” (*n* = 41; 37.3%).

**TABLE 2:** Purpose for using generative AI.

Purpose	*N* (%)
Generating ideas for research questions or hypotheses	18 (16.4%)
Drafting or structuring research papers, abstracts, or grants	41 (37.3%)
Proofreading or improving grammar and writing clarity	80 (72.7%)
Translating text into other languages	27 (24.5%)
Adapting content for different audiences (e.g., lay summaries, presentations)	41 (37.3%)
Extracting study characteristics or results from articles	14 (12.7%)
Assisting with citation or reference formatting	27 (24.5%)
Writing or debugging code for data analysis	46 (41.8%)
Performing qualitative data coding or thematic analysis	6 (5.5%)
Creating tables, figures, or visualizations from data	16 (14.5%)
Assisting with data cleaning or preparation	12 (10.9%)
Drafting ethics applications or participant materials	15 (13.6%)
Writing professional emails or research correspondence	45 (40.9%)
Generating PowerPoint slides or research posters	17 (15.5%)
Preparing reviewer responses or revision letters	22 (20.0%)
Converting text to speech for presentations, training modules, or accessibility	2 (1.8%)
Transcribing interviews, focus groups, or team meetings	8 (7.3%)
Creating synthetic datasets for testing analysis pipelines	2 (1.8%)
Designing customized stimuli for psychological or behavioral experiments	4 (3.6%)

#### 3.3.4 Verification Efforts

Generative AI users were asked to indicate how often they took steps to verify the outputs generated by AI tools before using them for research. Sixty‐nine participants (62.7%) responded “always,” 33 (30.0%) responded “often,” three each (2.7%) responded “sometimes” or “occasionally,” and one each (0.9%) responded “only once or twice” or “never.”

#### 3.3.5 Generative AI Disclosure

When asked if they had disclosed their use of generative AI in research, 75 (68.2%) responded “no” and 35 (31.8%) responded “yes.” Reasons for non‐disclosure, in order of endorsement, were that it was used in a minor way that did not seem worth disclosing (*n* = 45; 60%), not thinking it was necessary (*n* = 42; 56.0%), unsure how or where to disclose (*n* = 14; 18.7%), journal/conference/funder did not require disclosure (*n* = 13; 17.3%), concern about how disclosure would be perceived (*n* = 12; 16.0%), and did not consider it until after publication (*n* = 2; 2.7%). Thirteen participants (17.3%) selected “other,” with six indicating (5.4%) that they had not published any work yet that involved generative AI assistance.

#### 3.3.6 Generative AI Usefulness in Eating Disorder Research

When asked whether the use of generative AI had improved their ability to conduct eating disorder research, 68 (61.8%) responded “yes,” 24 (21.8%) responded “no,” and 18 (16.4%) responded “unsure.”

### 3.4 Generative AI Use in Peer Review

Of those that had reported using generative AI for research (*n* = 110), 23 (20.9%) indicated that they had used these tools in peer review while 87 (79.1%) indicated that they had not. Nearly three‐quarters reported using generative AI tools for peer review “only once or twice” (*n* = 7; 30.4%) or “occasionally” (*n* = 9; 39.1%), while three respondents each reported using them “sometimes” (13%) or “often” (13%), and one (4.3%) reported “always” using these tools for review tasks. More than half (*N* = 13; 56.5%) reported using generative AI to proofread or polish their review, while one‐third (*n* = 8; 34.8%) reported using it to help construct written paragraphs based on key bullet points provided. Two participants (8.7%) used generative AI to draft full submissions from uploaded content, which they then reviewed and edited. No participant reported relying primarily on generative AI to generate a full draft with minimal or no human oversight.

The full sample was asked whether they thought they had received peer review feedback that was AI‐generated. Twenty‐four (15.2%) responded “yes,” 105 (66.5%) responded “no” and 29 (18.4%) responded “unsure.”

### 3.5 Author Endorsement of Generative AI Use by Editors and Reviewers

Participants were presented with a list of potential tasks that generative AI could assist journal editors and peer reviewers with and were asked to select those they would feel comfortable with. The most frequently selected task was “detecting plagiarism or duplicated content” (*n* = 121; 76.6%), followed by “assisting editors in identifying reviewers” (*n* = 99; 62.7%), “translating reviewer/editor comments into clearer language” (*n* = 72; 45.6%), “evaluating the overlap or a submitted paper relative to existing literature” (*n* = 65; 41.1%), “crafting decision letters based on reviewer input” (*n* = 52; 32.9%), “assisting reviewers in drafting reviews with human oversight” (*n* = 43; 26.6%), and then “generating full reviews and making recommendations to editors” (*n* = 6; 3.8%). Sixteen participants (10.1%) stated that they would not be comfortable for generative AI to be used in any part of the submission or review process.

### 3.6 Perceived Benefits and Limitations of Generative AI for Eating Disorder Research

Table 3 presents the percentage of participants endorsing proposed benefits and limitations of using generative AI for eating disorder research. Benefits that received the highest percentage of endorsement were improved efficiency of research tasks (*n* = 93; 58.9%), supporting non‐native English speakers (e.g., editing, translation) (*n* = 83; 52.5%), and supporting code generation or data analysis (*n* = 79; 50.0%). Limitations that received the highest percentage of endorsement were the potential for misinformation (*n* = 150; 94.9%), ethics related to plagiarism, originality, and confidentiality (*n* = 139; 88.0%), and reduced critical thinking or an over‐reliance on AI tools (*n* = 127; 80.4%).

**TABLE 3:** Benefits and limitations of generative AI in eating disorder research.

Benefits	*N* (%)
Improves efficiency across research‐related tasks, including writing and administration	93 (58.9%)
Improves the overall quality of the research and advances scientific discovery of eating disorders	15 (9.5%)
Helps eating disorder researchers without English as a first language (through editing or translation).	83 (52.5%)
Enhances creativity and hypothesis generation	28 (17.7%)
Supports code generation or data analysis	79 (50.0%)
Supports communication with diverse audience	49 (31.0%)
Facilitates cross‐disciplinary integration (e.g., linking insights from psychology, medicine, nutrition, or data science)	21 (13.3%)
Improves access to research support for early‐career researchers or those in under‐resourced settings	46 (29.1%)
Assists with literature reviews or rapid evidence synthesis	65 (41.1%)
Other	16 (10.1%)
There are no benefits	3 (1.9%)

Limitations	*N* (%)
Potential for inaccurate or misleading information	150 (94.9%)
Ethical concerns around plagiarism, originality, and authorship	139 (88.0%)
Difficulty detecting hallucinations (i.e., errors that have the appearance of veracity) or fabricated references	105 (66.5%)
Reduced critical thinking or over‐reliance on generative AI outputs	127 (80.4%)
Privacy or data security risks when uploading sensitive eating disorder‐related materials	86 (54.4%)
Lack of transparency in how generative AI systems generate content	97 (61.4%)
Technical limitations (e.g., inability to handle large data sets)	31 (19.6%)
Unequal access to high‐performing generative AI tools across researchers	52 (32.9%)
Risk of generating content that reinforces eating disorder stereotypes	79 (50.0%)
Limited understanding of the complexity and nuance of eating disorders	88 (55.7%)
Lack of clear guidelines for responsible use in eating disorder contexts	103 (65.2%)
Other	15 (9.5%)
There are no limitations	0 (0%)

### 3.7 Anticipated Influence of Generative AI on the Field

Most participants agreed or strongly agreed that generative AI will continue to improve access to language‐related research support for non‐native English‐speaking researchers (87%), enhance innovation in digitally delivered treatments (65%), and accelerate the pace of research outputs in the field (55%). However, most also agreed or strongly agreed that generative AI will increase the risk of inaccurate findings in the field (72%), complicate the ethical landscape for developing and testing eating disorder interventions (60%), and increase research volume but reduce the depth of outputs in the field (59%). See Table 4.

**TABLE 4:** Agreement ratings for generative AI's impact in eating disorder research over the next 5 years.

Statement	Strongly disagree	Disagree	Neither agree nor disagree	Agree	Strongly agree
Generative AI will accelerate the pace of research and publication in the eating disorder field.	4 (2.5%)	12 (7.6%)	54 (34.2%)	63 (39.9%)	25 (15.8%)
Generative AI will improve access to language‐related research support for non‐native English‐speaking researchers (e.g., assistance with writing, editing, or translating research materials).	1 (0.6%)	1 (0.6%)	18 (11.4%)	106 (67.1%)	32 (20.3%)
Generative AI will enhance innovation in digital interventions for eating disorders (e.g., chatbots, adaptive tools).	5 (3.2%)	12 (7.6%)	39 (24.7%)	73 (46.2%)	29 (18.4%)
Generative AI will broaden opportunities for interdisciplinary collaboration among eating disorder researchers (e.g., between data science, psychology, and medicine).	6 (3.8%)	34 (21.5%)	60 (38.0%)	47 (29.7%)	11 (7.0%)
Generative AI will increase the risk of inaccurate or oversimplified findings in eating disorder research.	2 (1.3%)	8 (5.1%)	33 (20.9%)	73 (46.2%)	42 (26.6%)
Generative AI will widen existing inequalities between well‐resourced and under‐resourced researchers or institutions.	5 (3.2%)	28 (17.7%)	63 (39.9%)	48 (30.4%)	14 (8.9%)
Generative AI will complicate the ethical landscape for developing and testing eating disorder interventions.	4 (2.5%)	13 (8.2%)	46 (29.1%)	62 (39.2%)	33 (20.9%)
Generative AI will increase the volume but reduce the depth of research outputs in the field (e.g., originality, theoretical insight, or methodological rigor).	4 (2.5%)	21 (13.3%)	40 (25.3%)	63 (39.9%)	30 (19.0%)
Generative AI will raise new concerns about the use of sensitive or potentially triggering content in ED‐related materials (e.g., specific references to weight, calories, or detailed eating behaviors such as purging or restriction methods).	7 (4.4%)	17 (10.8%)	58 (36.7%)	49 (31.0%)	27 (17.1%)
Generative AI will have little to no impact on the field of eating disorder research.	80 (50.6%)	59 (37.3%)	18 (11.4%)	1 (0.6%)	0 (0%)

## 4 Discussion

### 4.1 Summary of Findings

We found evidence of widespread use of generative AI tools among a sample of 158 eating disorder researchers: 70% reported using generative AI to assist with research‐related tasks, most commonly for proofreading written texts but also for code support, drafting professional emails and papers, and adapting research reports for different audiences. Encouragingly, 9 in 10 users reported either always or often verifying the outputs produced by these tools, which is important given that generative AI has been shown to produce fabricated but plausible‐sounding content (Alkaissi and McFarlane 2023; Huang et al. 2025). Indeed, concerns about the accuracy of AI‐generated content were the most frequently endorsed reason for non‐use (71%). Despite widespread use, two‐thirds of users reported not disclosing their use of generative AI in research papers, primarily because they felt it was used in a minor way that did not warrant disclosure, did not believe disclosure was necessary, or were unsure how or where to disclose it. This suggests a gap between actual use and current disclosure patterns, highlighting the potential need for journal operating systems to incorporate prompts that explicitly ask authors to indicate whether, and at what level, generative AI was used during the preparation of the manuscript.

This lack of consistent disclosure aligns with broader uncertainty in the research community about where to seek guidance on responsible AI use. Recent global data demonstrate that a majority of researchers would like publishers to take a more proactive role in the use of AI in academic publishing (Wiley 2025). Specifically, 70% of researchers surveyed want publishers to provide clear guidelines on acceptable AI use in scholarly publishing, and over two‐thirds want help avoiding common limitations, such as bias and inaccuracy (Wiley 2025). Furthermore, our findings align with surveys of researchers in other disciplines that similarly highlight high rates of AI use alongside uncertainty around disclosure and ethics (Linardon et al. 2025; Van Noorden and Perkel 2023). While many of these issues, such as accuracy, plagiarism, and transparency, are shared across academia, our work emphasizes considerations uniquely salient for eating disorders and mental health research, including the risks of reinforcing stigma and the need for careful handling of sensitive participant data.

Findings also generated novel insights into current practices and perspectives of generative AI in the peer review and publishing process. Only 1 in 5 users reported engaging with generative AI for peer review tasks, which likely reflects ongoing uncertainty about the appropriateness of AI use in peer evaluation and concerns about maintaining the integrity of the review process (Donker 2023; Hosseini and Horbach 2023). Even so, the use of generative AI in peer review was infrequent and generally limited in scope, with most respondents indicating they used these tools to assist with presentation‐related tasks (e.g., proofreading or constructing paragraphs from bullet points) with full human oversight. Beyond their own use, participants also shared views on the role of generative AI in peer review and editorial decision‐making more broadly. When presented with a list of potential AI‐assisted tasks, the majority expressed comfort with its use for administrative or evaluative support, particularly for detecting plagiarism or duplicated content (76.6%) and assisting editors in identifying reviewers (62.7%). In contrast, 4% supported the use of generative AI for fully generating reviews and making editorial recommendations, and 10% indicated discomfort with its use at any stage of the submission or review process.

Although findings show that generative AI is used by a significant proportion of researchers in our field, enthusiasm for these tools is tempered by recognition of their inherent limitations and the potential for adverse impacts on eating disorder research. The majority of respondents acknowledged significant limitations of generative AI, including its potential to produce inaccurate content, ethical concerns related to originality and authorship, reduced critical thinking, and risks to data privacy. Additional concerns specific to the eating disorder field noted by participants included the reinforcement of harmful stereotypes, the inability of AI systems to grasp the complexity and nuance of eating disorders, and a lack of clear guidelines for responsible use in this context. Reflecting these concerns, a large proportion of participants anticipated that generative AI could negatively influence the future of eating disorder research by increasing the risk of oversimplified findings, complicating the ethical landscape for intervention development, and contributing to a higher volume but reduced depth of scholarly outputs. While benefits of generative AI were also recognized by many (e.g., improved task efficiency, supporting non‐native English speakers), these findings reinforce the importance of developing clear, context‐sensitive standards that promote responsible use while safeguarding the integrity of eating disorder research.

### 4.2 Limitations

It is important to acknowledge the limitations of this survey. First, participants self‐selected into the study, which may have introduced sampling bias, as those with greater interest in or familiarity with generative AI may have been more likely to participate. This may explain why a significant proportion of the sample was research students or reported being earlier in their career. Second, we relied on self‐report data, which may limit the depth of insight into participants' experiences and perspectives. While open‐ended response options were included among the items, the absence of opportunities for clarification or follow‐up may have constrained the richness of responses. However, the anonymous format may have encouraged more candid disclosure, particularly regarding the extent and nature of AI use. Third, the sample may not be fully representative of the global eating disorder research community, as recruitment was limited to specific professional networks and journals, potentially underrepresenting perspectives from regions or career stages less connected to these channels. Fourth, survey items were developed by the IJED Executive Committee based on prior generative AI survey research and their collective expertise. Although this ensured contextual relevance, we did not follow a formal survey design framework, and the potential for unintended bias in item framing or emphasis cannot be excluded.

### 4.3 Recommendations for Generative AI Use in Eating Disorders

These findings offer valuable insights into the current uses, perceptions, and concerns surrounding generative AI among eating disorder researchers. Drawing on these data and referring to recommendations promulgated by an expert panel of researchers and leaders in higher education and science publishing and communication (Blau et al. 2024), we propose our recommendations (summarized in Table 5) for the responsible use of generative AI by authors, reviewers, and editors in the field. While these recommendations are intended to inform IJED's editorial practices, we also hope they will be embraced and adapted by other journals in our field to promote consistent, ethical, and transparent standards.

**TABLE 5:** Recommendations for generative AI use by the *International Journal of Eating Disorders*.

Personnel	Category	Recommendations and practices	Example(s)
Authors	What you can do now	Disclose all AI use in manuscripts (methods, acknowledgments, or disclosure statement). Use AI for low‐level support tasks only (proofreading, formatting, debugging code, language correction). Verify outputs against source data and professional standards; prompt for cultural diversity and bias reduction.	Proofreading: grammar/spelling only. Prompts: “Provide examples across diverse genders and body sizes.”
	What to avoid	Do not use AI to write the main text or generate analytic content. Do not upload identifiable/sensitive data. Do not rely on AI for clinical/diagnostic content or lived experience perspectives. Do not list AI as an author. Do not use AI‐generated text verbatim due to risks of plagiarism and unacknowledged reuse.	
	Areas for further development/research	Research on prompt engineering for bias mitigation (i.e., correct wording, degree of specificity, inclusivity). Research on hallucinations, misinformation, and risk mitigation (extent of these errors, domains most likely observed). Clear privacy and data governance architecture. Cultural considerations in outputs.	
Reviewers	What you can do now	Use AI only for minor support tasks (proofreading, organizing comments). Disclose AI use and the extent of its use in confidential comments to editors and to authors. Review the terms of use before employing any AI platform for review purposes	Proofreading draft review text.
	What to avoid	Do not use AI to evaluate methodology, interpret findings, or make editorial recommendations. Do not upload manuscript text into AI systems.	
	Areas for further development/research	Best practices for secure AI‐assisted reviewing (protocols to ensure confidentiality, prevent manuscript text from being stored or reused, and clarify acceptable reviewer use of AI). Prompt design to reduce bias in reviews (encouraging balanced language, inclusion of diverse perspectives, and avoiding stigmatizing terms).	
Editors	What you can do now	May use AI tools with human oversight for reviewer selection, plagiarism detection, or language support. Retain human responsibility for all editorial decisions.	AI flagging overlap, editor decides on action.
	What to avoid	Do not use AI to evaluate manuscripts, generate decision letters, or summarize reviews without human oversight.	
	Areas for further development/research	Consider development of secure, closed AI platforms for editorial tasks (journal‐ or publisher‐specific systems that ensure confidentiality and prevent data reuse). Regular review and updating of guidelines based on new data, new perspectives (inclusive working group, including lived experience persons, AI developers, marginalized groups), and new generative AI software.	

#### 4.3.1 For Authors

Authors are encouraged to approach the use of generative AI in eating disorder research with transparency, caution, and integrity. Any use of generative AI tools during manuscript preparation (such as for editing, summarizing, coding support, or figure generation) should be clearly disclosed. Appropriate places for disclosure include the methods section, acknowledgments, or a dedicated disclosure statement, wherever authors deem most appropriate. An example disclosure statement may include:

The authors used the generative artificial intelligence platform [list the platform] to assist with [briefly describe tasks, for example, editing, coding support, language correction/translation, etc.] during the preparation of this manuscript. We confirm that all AI‐assisted content was carefully reviewed and edited by the authors to ensure accuracy and appropriateness.

To enhance transparency and standardize reporting, we recommend that all submitted manuscripts include a definitive AI disclosure statement, indicating clearly whether generative AI was or was not used during manuscript preparation. Journal websites should support this by implementing a mandatory prompt or checkbox at submission, with detailed follow‐up options on how AI was used.

When used, we recommend that generative AI tools be limited to supporting routine or low‐level tasks (e.g., proofreading, formatting, debugging code) rather than generating original content or conducting deeper analytic work. Furthermore, we do not recommend that authors use or rely on generative AI to write any part of the main text in the manuscript. Relying on AI as a primary content creator risks undermining the originality, theoretical nuance, and critical insight expected in scholarly research. AI outputs must be reviewed for language that could reinforce harmful stereotypes or stigma. Authors should never rely on AI to represent the lived experiences of individuals with eating disorders. Where available, authors should also familiarize themselves with platform settings that allow opting out of data sharing and ensure these are enabled to protect research integrity and participant confidentiality.

In all cases, authors must critically evaluate and fact‐check AI‐generated content, ensuring that inaccurate, fabricated, or oversimplified outputs are not included in published work. Any content generated through AI remains the responsibility of the author, and we discourage the use of AI‐generated text verbatim due to the risk of plagiarism, replication of training data, or unacknowledged reuse (Flanagin et al. 2023). Authors should also be aware that the specificity and framing of prompts strongly influence AI outputs, and should take care to design prompts thoughtfully, review outputs critically, and avoid prompts that could elicit biased or inappropriate responses. Per the Committee on Publication Ethics (COPE) and the International Committee of Medical Journal Editors (ICMJE) 2023 guidelines, authorship must not be attributed to AI tools, as these systems cannot take responsibility for the work nor provide informed consent.

#### 4.3.2 For Reviewers

Peer reviewers must approach the use of generative AI with caution, transparency, and a strong commitment to confidentiality. If generative AI is used in any part of the peer review process, reviewers remain fully responsible for the accuracy, quality, and integrity of the submitted review content. We recommend that generative AI tools be used only for minor support tasks (e.g., proofreading or reorganizing reviewer comments), not for drafting reviews, evaluating scientific merit, or making editorial recommendations.

All AI‐assisted content must be carefully and critically reviewed and edited to ensure accuracy, fairness, and professionalism. Reviewers should remain alert to potential biases, oversimplifications, or misleading interpretations generated by AI systems and must apply their own domain expertise and ethical judgment to ensure that outputs align with scholarly standards. Generative AI should never be relied upon to assess methodological rigor, interpret findings, or influence evaluative recommendations, as these tasks require human expertise and critical reasoning beyond the capabilities of current AI tools. This recommendation is specific to peer reviewers, as evaluating methodology or conclusions involves judging others' work—responsibilities that must rest with human experts—whereas authors remain accountable for the accuracy and integrity of their own interpretations.

Maintaining the confidentiality of the peer review process is critical. Consistent with the Committee on Publication Ethics (COPE) guidelines, the IJED does not permit reviewers to input any portion of a manuscript under review into generative AI systems (Wiley n.d.). This is because many AI systems (e.g., Open AI's ChatGPT) may store and reuse user input to further develop and improve their services, unless users explicitly opt out. Doing so poses a serious risk to the security and confidentiality of unpublished research and constitutes an ethics violation. Reviewers are strongly encouraged to review the terms of use for any AI platform they intend to use.

If a reviewer uses generative AI in any capacity, this use should at a minimum be disclosed in the confidential comments to the editor. The editor may then decide whether disclosure to the author is warranted, depending on the nature and extent of AI use. This level of transparency is necessary, as a sizable minority of authors suspected they have received AI‐generated reviews.

#### 4.3.3 For Editors

At the IJED, editors do not currently use generative AI tools for evaluating manuscripts, drafting decision letters, or summarizing reviewer comments. Editorial decisions remain grounded in expert human judgment and domain knowledge. However, the editorial team and publisher do make use of advanced technologies that incorporate elements of AI that help identify suitable reviewers, flag potential plagiarism or overlapping content, or assist with language correction for editors for whom English is not their first language. In the future, we may also adopt tools that can help detect whether a manuscript includes content generated by AI. In all instances, these technologies are used to support, not replace, editorial decision‐making. Editors retain full responsibility for all editorial actions and exercise human oversight throughout the review and publication process carefully.

### 4.4 Concluding Remarks

As generative AI continues to evolve, it presents both opportunities and challenges for the eating disorder research community. By foregrounding transparency, critical oversight, and ethical responsibility, researchers, reviewers, and editors can harness the benefits of these tools while safeguarding the integrity and nuance required in our field. The present findings and recommendations offer a foundation for guiding responsible AI use as we move toward a more technologically integrated research landscape. Given the rapid pace of generative AI development, these guidelines should be reviewed and updated at least annually to ensure they remain current and aligned with emerging technologies, ethical standards, and field needs. Looking ahead, there may be value in developing closed, secure AI platforms tailored for the eating disorder (or broader) research community, which could support field‐wide analyses that extend beyond the capacity of individual researchers.

## Author Contributions

**Jake Linardon:** conceptualization, writing – original draft, formal analysis, writing – review and editing. **Jennifer J. Thomas:** conceptualization, methodology, writing – review and editing. **Scott J. Crow:** conceptualization, writing – review and editing. **Ata Ghaderi:** conceptualization, writing – review and editing. **Anja Hilbert:** conceptualization, writing – review and editing. **Kelly L. Klump:** conceptualization, writing – review and editing. **Tracey D. Wade:** conceptualization, writing – review and editing. **B. Timothy Walsh:** conceptualization, writing – review and editing. **Ruth Weissman:** conceptualization, writing – original draft, writing – review and editing, methodology, supervision.

## Conflicts of Interest

All authors, except J.J.T, of this submission are on the Executive Committee for the *International Journal of Eating Disorders*.

## Supporting information


**Data S1:** Supporting Information.

## Data Availability

The data that support the findings of this study are available from the corresponding author upon reasonable request.

## References

[eat24543-bib-0001] Alkaissi, H. , and S. I. McFarlane . 2023. “Artificial Hallucinations in ChatGPT: Implications in Scientific Writing.” Cureus 15, no. 2: e35179. 36811129 10.7759/cureus.35179 PMC9939079

[eat24543-bib-0002] Andersen, J. P. , L. Degn , R. Fishberg , et al. 2025. “Generative Artificial Intelligence (GenAI) in the Research Process – A Survey of Researchers' Practices and Perceptions.” Technology in Society 81: 102813. 10.1016/j.techsoc.2025.102813.

[eat24543-bib-0003] Blau, W. , V. G. Cerf , J. Enriquez , et al. 2024. “Protecting Scientific Integrity in an Age of Generative AI.” Proceedings of the National Academy of Sciences of the United States of America 121: e2407886121. 38771193 10.1073/pnas.2407886121 PMC11145223

[eat24543-bib-0004] Donker, T. 2023. “The Dangers of Using Large Language Models for Peer Review.” Lancet Infectious Diseases 23, no. 7: 781. 10.1016/S1473-3099(23)00290-6 37178707

[eat24543-bib-0005] Ertel, W. 2024. Introduction to Artificial Intelligence. Springer Nature.

[eat24543-bib-0006] Flanagin, A. , J. Kendall‐Taylor , and K. Bibbins‐Domingo . 2023. “Guidance for Authors, Peer Reviewers, and Editors on Use of AI, Language Models, and Chatbots.” JAMA 330, no. 8: 702–703. 37498593 10.1001/jama.2023.12500

[eat24543-bib-0007] Hosseini, M. , and S. P. Horbach . 2023. “Fighting Reviewer Fatigue or Amplifying Bias? Considerations and Recommendations for Use of ChatGPT and Other Large Language Models in Scholarly Peer Review.” Research Integrity and Peer Review 8, no. 1: 4. 37198671 10.1186/s41073-023-00133-5 PMC10191680

[eat24543-bib-0008] Huang, L. , W. Yu , W. Ma , et al. 2025. “A Survey on Hallucination in Large Language Models: Principles, Taxonomy, Challenges, and Open Questions.” ACM Transactions on Information Systems 43, no. 2: 1–55.

[eat24543-bib-0009] Leung, T. I. , T. de Azevedo Cardoso , A. Mavragani , and G. Eysenbach . 2023. “Best Practices for Using AI Tools as an Author, Peer Reviewer, or Editor.” Journal of Medical Internet Research 25: e51584 Toronto, Canada. 37651164 10.2196/51584 PMC10502596

[eat24543-bib-0010] Linardon, J. , M. Messer , C. Anderson , et al. 2025. “Role of Large Language Models in Mental Health Research: An International Survey of Researchers' Practices and Perspectives.” BMJ Mental Health 28, no. 1: e301787. 10.1136/bmjment-2025-301787 PMC12164621 40514050

[eat24543-bib-0011] Luo, X. , Y. C. Tham , M. Giuffrè , et al. 2025. “Reporting Guideline for the Use of Generative Artificial Intelligence Tools in MEdical Research: The GAMER Statement.” BMJ Evidence‐Based Medicine: 113825. 10.1136/bmjebm-2025-113825 PMC12703294 40360239

[eat24543-bib-0012] Lv, Z. 2023. “Generative Artificial Intelligence in the Metaverse Era.” Cognitive Robotics 3: 208–217.

[eat24543-bib-0013] Moy, L. 2023. “Guidelines for Use of Large Language Models by Authors, Reviewers, and Editors: Considerations for Imaging Journals.” Radiology 309: e239024. 37815449 10.1148/radiol.239024

[eat24543-bib-0014] Nicholas, D. , M. Swigon , D. Clark , et al. 2024. “The Impact of Generative AI on the Scholarly Communications of Early Career Researchers: An International, Multi‐Disciplinary Study.” Learned Publishing 37, no. 4: e1628.

[eat24543-bib-0015] Torous, J. , and C. Blease . 2024. “Generative Artificial Intelligence in Mental Health Care: Potential Benefits and Current Challenges.” World Psychiatry 23, no. 1: 1–2. 10.1002/wps.21148. 38214643 PMC10785974

[eat24543-bib-0016] Van Dalen, H. P. 2021. “How the Publish‐Or‐Perish Principle Divides a Science: The Case of Economists.” Scientometrics 126, no. 2: 1675–1694.

[eat24543-bib-0017] Van Noorden, R. , and J. M. Perkel . 2023. “AI and Science: What 1,600 Researchers Think.” Nature 621, no. 7980: 672–675. 37758894 10.1038/d41586-023-02980-0

[eat24543-bib-0018] Wiley . 2025. ExplanAItions: An AI study by Wiley. https://www.wiley.com/en‐au/ai‐study.

[eat24543-bib-0019] Wiley . n.d. “Review Confidentiality Policy.” https://authorservices.wiley.com/Reviewers/journal‐reviewers/tools‐and‐resources/review‐confidentiality‐policy.html.

