# Supplementary material for: Conducting Eating Disorder Research in the Era of Generative AI: Researcher Perspectives and Guidelines From the International Journal of Eating Disorders
Source: Int J Eat Disord. 2025 Sep 7;58(12):2306–16. doi: 10.1002/eat.24543 (PMC12703213; doi:10.1002/eat.24543)
Supplement: Supplementary file 1 — Data S1: Supporting Information. [file EAT-58-2306-s001.docx]

AI in eating disorder research survey

Q3 What is your age in years (please write one whole number only [e.g., 42])?

________________________________________________________________

________________________________________________________________

________________________________________________________________

________________________________________________________________

________________________________________________________________

Q4 What gender do you identify with?

- Male (1)
- Female (2)
- Non-binary (3)
- Prefer not to say (4)
- Other (specify) (5) __________________________________________________

Q5 What country do you currently live in (please write the country in full [e.g., United States])?

________________________________________________________________

________________________________________________________________

________________________________________________________________

________________________________________________________________

________________________________________________________________

Q6 What is your highest academic qualification?

- Bachelor's degree or equivalent (1)
- Master's degree or equivalent (2)
- PhD/Doctorate degree (3)
- MD (4)
- Other (specify) (5) __________________________________________________

Q7 Select the current job title/role that best describes you?

- Research Student (PhD/doctoral) (1)
- Post-Doctoral Researcher (2)
- [Senior] Research Fellow (3)
- Lecturer/Assistant Professor (4)
- Associate Professor (5)
- Professor (6)
- Researcher-Scientist (7)
- Clinician-Scientist (8)
- Other (specify) (9) __________________________________________________

Q8 Approximately how many years of research experience do you have (enter a whole number only [e.g., 6])?

________________________________________________________________

________________________________________________________________

________________________________________________________________

________________________________________________________________

________________________________________________________________

Q9 Approximately how many peer-reviewed publications have you authored or co-authored?

- 1-20 (1)
- 21-40 (2)
- 41-60 (3)
- 61-80 (4)
- 81-100 (5)
- 101-150 (6)
- >150 (7)
- Prefer not to say (8)
- Not sure (9)

Q10 What is your primary focus within eating disorder research (select all that apply)?

- Treatment/early intervention (1)
- Prevention (2)
- Neurobiology/neuroimaging (3)
- Genetics (4)
- Assessment & diagnosis (5)
- Epidemiology (6)
- Etiology, risk factors and correlates (7)
- Health services and implementation science (8)
- Policy and advocacy (9)
- Other (specify) (10) __________________________________________________

Q11 The following questions ask about your experience with and perspectives towards generative artificial intelligence ("Gen AI"). Gen AI refers to AI systems that can create new content such as text, images, audio, or code. These tools are trained on large datasets and can generate human-like responses or original outputs based on a prompt. Common examples include ChatGPT, DALL·E, Midjourney, and GitHub Copilot. Gen AI can assist with tasks like summarizing articles, drafting emails, creating figures, writing code, and generating research ideas.

Q12 Have you ever used a Gen AI tool to assist with any aspect of your own research process? We’re referring to using Gen AI as a tool to support your own research activities – not studying or testing Gen AI systems themselves.

- Yes (1)
- No (2)

Start of Block: Yes respondents

Q14 Which Gen AI tools have you used for your research (select all that apply)?

- ChatGPT (OpenAI) (1)
- Google Gemini (formerly Bard) (2)
- Claude (Anthropic) (3)
- Perplexity AI (4)
- Mistral (5)
- LLaMA (Meta) (6)
- GitHub Copilot (powered by Codex/OpenAI) (7)
- DALL·E (OpenAI) (8)
- Microsoft Copilot (in Word, PowerPoint, Excel) (9)
- Other (specify) (10) __________________________________________________

Q15 How frequently do you use Gen AI tools for research-related tasks?

- Daily (1)
- Weekly (2)
- Monthly (3)
- Occasionally (less than once a month) (4)
- Only used once or twice (5)

Q16 For what purposes have you used Gen AI in your research (Select all that apply)?

- Generating ideas for research questions or hypotheses (1)
- Drafting or structuring research papers, abstracts, or grants (2)
- Proofreading or improving grammar and writing clarity (3)
- Translating text into other languages (4)
- Adapting content for different audiences (e.g., lay summaries, presentations) (5)
- Extracting study characteristics or results from articles (6)
- Assisting with citation or reference formatting (7)
- Writing or debugging code for data analysis (8)
- Performing qualitative data coding or thematic analysis (9)
- Creating tables, figures, or visualizations from data (10)
- Assisting with data cleaning or preparation (11)
- Drafting ethics applications or participant materials (12)
- Writing professional emails or research correspondence (13)
- Generating PowerPoint slides or research posters (14)
- Preparing reviewer responses or revision letters (15)
- Converting text to speech for presentations, training modules, or accessibility (16)
- Transcribing interviews, focus groups, or team meetings (17)
- Creating synthetic datasets for testing analysis pipelines (18)
- Designing customized stimuli for psychological or behavioral experiments (19)
- None of these (please specify) (20) __________________________________________________

Q17 How often do you take steps to verify the accuracy of Gen AI content before using it in your research? (for example, checking sources, confirming factual accuracy, reviewing AI-edited text for errors)

- Never (1)
- Only once or twice (2)
- Occasionally (in a few instance) (3)
- Sometimes (about half of the time) (4)
- Often (most of the time) (5)
- Always (in every instance) (6)

Q19 Have you ever used Gen AI to assist with any peer review or evaluation tasks (e.g., reviewing manuscripts, grants, or conference abstracts)?

- Yes (1)
- No (2)

Start of Block: Yes peer rev

Q20 How frequently have you used Gen AI to assist with peer review or evaluation tasks (e.g., manuscripts, grants, abstracts)?

- Only once or twice (1)
- Occasionally (a few instances) (2)
- Sometimes (used in about half of my reviews) (3)
- Often (used in most reviews) (4)
- Always (used in all my reviews) (5)

Yes peer rev To what extent, on average, do you rely on Gen AI when using it for peer review or evaluation tasks?

- I only use it to proofread or polish my review before submitting (1)
- I use it to help construct written paragraphs based on key points I provide it (2)
- I upload the manuscript, grant, or abstract and use Gen AI to generate a full draft, which I carefully review and edit before submitting (3)
- I upload the manuscript, grant, or abstract and rely almost entirely on Gen AI to generate a full review, with minimal or no human oversight (4)
- Other (specify) (5) __________________________________________________

Disclosed Have you ever disclosed your use of Gen AI in your research (e.g., in a methods section, acknowledgments, author list)?

- No (1)
- Yes (2)

End of Block: Yes respondents

Start of Block: No disclosed

Q23 Why have you not disclosed your use of Gen AI in your research? (select all that apply)

- I didn’t think disclosure was necessary (1)
- I wasn’t sure how or where to disclose (2)
- The journal/conference/funder did not require disclosure (3)
- I was concerned about how disclosure would be perceived (4)
- I used it in a very minor way that didn’t seem worth reporting (5)
- I didn’t consider it until after submission/publication (6)
- Other (specify) (7) __________________________________________________

Q24 Has using generative AI improved your ability to conduct eating disorder research (e.g., by increasing your efficiency, supporting tasks outside your expertise, or enhancing your research process)?

- Yes (1)
- Unsure (2)
- No (3)

Q13 Why have you not used Gen AI tools for your research? (select all that apply)

- I’m not familiar with Gen AI tools or haven’t considered using them for research purposes (1)
- Limited time or interest in learning Gen AI tools (2)
- Concerns about the accuracy or reliability of Gen AI output (3)
- Concerns about plagiarism or academic misconduct (4)
- Lack of access to Gen AI tools I would use (due to paywalls or licensing issues) (5)
- Concerned that use of Gen AI could undermine originality and creativity (6)
- Concerns about data privacy and confidentiality (7)
- Lack of policies or unclear guidelines around Gen AI use (8)
- Departmental, School or peer discouragement (9)
- Concerns about reputational risks if use is perceived as inappropriate (10)
- Preference for human-led thinking and writing (11)
- Other (specify) (12) __________________________________________________

Q25 In your view, what are the current **benefits** of Gen AI for eating disorder research, regardless if you have or have not used these tools? (Select all that apply)

- Improves efficiency across research-related tasks, including writing and administration (1)
- Improves the overall quality of the research and advances scientific discovery of eating disorders (2)
- Helps eating disorder researchers without English as a first language (through editing or translation). (3)
- Enhances creativity and hypothesis generation (4)
- Supports code generation or data analysis (5)
- Supports communication with diverse audience (6)
- Facilitates cross-disciplinary integration (e.g., linking insights from psychology, medicine, nutrition, or data science) (7)
- Improves access to research support for early-career researchers or those in under-resourced settings (8)
- Assists with literature reviews or rapid evidence synthesis (9)
- Other (specify) (10) __________________________________________________
- There are no benefits (11)

| Page Break |  |
| --- | --- |

Q26 In your view, what are the current **limitations** of Gen AI for eating disorder research, regardless if you have or have not used these tools? (Select all that apply)

- Potential for inaccurate or misleading information (1)
- Ethical concerns around plagiarism, originality, and authorship (2)
- Difficulty detecting hallucinations (i.e., errors that have the appearance of veracity) or fabricated references (3)
- Reduced critical thinking or over-reliance on gen AI outputs (4)
- Privacy or data security risks when uploading sensitive eating disorder-related materials (5)
- Lack of transparency in how gen AI systems generate content (6)
- Technical limitations (e.g., inability to handle large data sets) (7)
- Unequal access to high-performing gen AI tools across researchers (8)
- Risk of generating content that reinforces eating disorder stereotypes (9)
- Limited understanding of the complexity and nuance of eating disorders (10)
- Lack of clear guidelines for responsible use in eating disorder contexts (11)
- Other (specify) (13) __________________________________________________
- There are no limitations (14)

End of Block: ALL PARTICIPANTS

Start of Block: All participants

Q27 Have you ever suspected that peer review comments you received were composed using Gen AI?

- No (1)
- Unsure (2)
- Yes (3)

| Page Break |  |
| --- | --- |

Q28 When you submit a manuscript to a journal, which of the following uses of Gen AI by journal editors or peer reviewers would you be comfortable with? (select all that apply)

- Detecting plagiarism or duplicated content (1)
- Evaluating the overlap or novelty of a submitted paper relative to existing literature (2)
- Assisting editors in identifying suitable peer reviewers (3)
- Assisting peer reviewers in drafting reviews (with human oversight) (4)
- Generating full peer reviews and making recommendations to editors (5)
- Crafting editorial decision letters based on reviewer input (6)
- Translating reviewer or editorial comments into clearer language (7)
- I would not be comfortable with Gen AI being used in any part of the manuscript or peer review process (8)

Q31 Please rate the extent to which you agree with the following statements about how Gen AI may impact the field of eating disorder research over the next 5 years:

|  | Strongly disagree (1) | Disagree (2) | Neither agree nor disagree (3) | Agree (4) | Strongly agree (5) |
| --- | --- | --- | --- | --- | --- |
| Generative AI will accelerate the pace of research and publication in the eating disorder field. (1) |  |  |  |  |  |
| Generative AI will improve access to language-related research support for non-native English-speaking researchers (e.g., assistance with writing, editing, or translating research materials). (2) |  |  |  |  |  |
| Generative AI will enhance innovation in digital interventions for eating disorders (e.g., chatbots, adaptive tools). (3) |  |  |  |  |  |
| Generative AI will broaden opportunities for interdisciplinary collaboration among eating disorder researchers (e.g., between data science, psychology, and medicine). (4) |  |  |  |  |  |
| Generative AI will increase the risk of inaccurate or oversimplified findings in eating disorder research. (5) |  |  |  |  |  |
| Generative AI will widen existing inequalities between well-resourced and under-resourced researchers or institutions. (6) |  |  |  |  |  |
| Generative AI will complicate the ethical landscape for developing and testing eating disorder interventions. (7) |  |  |  |  |  |
| Generative AI will increase the volume but reduce the depth of research outputs in the field (e.g., originality, theoretical insight, or methodological rigor) (8) |  |  |  |  |  |
| Generative AI will raise new concerns about the use of sensitive or potentially triggering content in ED-related materials (e.g., specific references to weight, calories, or detailed eating behaviors such as purging or restriction methods) (9) |  |  |  |  |  |
| Generative AI will have little to no impact on the field of eating disorder research. (10) |  |  |  |  |  |
